# Supplementary material for: Evolution and development of fruits of Erycina pusilla and other orchid species
Source: PLoS One. 2023 Oct 10;18(10):e0286846. doi: 10.1371/journal.pone.0286846 (PMC10564159; doi:10.1371/journal.pone.0286846)
Supplement: S1 Fig — Cross-sections of developing E. pusilla (A-F) and P. equestris fruits (G-H), embedded in LR White and stained with toluidine blue. (A) Fruit 7 days after pollination (DAP). (B) Magnified part of the sterile valve at 2 weeks after pollination (WAP). Arrows indicate the dehiscence zone. Black boxes the exo-, meso- and endocarp layer. (C) Fruit of 2 WAP. (D) Fruit of 4 WAP. (E) Fruit of 16 WAP. (F) Magnified part of the sterile valve at 16 WAP. (G) Fruit of 120 DAP. (G) Magnified part of the sterile valve at 120 DAP; cell with thick blue stained wall part of vascular bundle (VB) indicates sclerenchyma fiber cap. Black arrows indicate the dehiscence zone. F, fertile valve; S, sterile valve; PT, pollen tube. Scale bar (C, K) = 0.2 mm, (D) = 0.1 mm, (E–I) = 1 mm, (J, G, H) = 0.5 mm. [Figs A-F from [31]; Figs G-H captured by Dewi Pramanik]. (DOCX) [file pone.0286846.s001.docx]

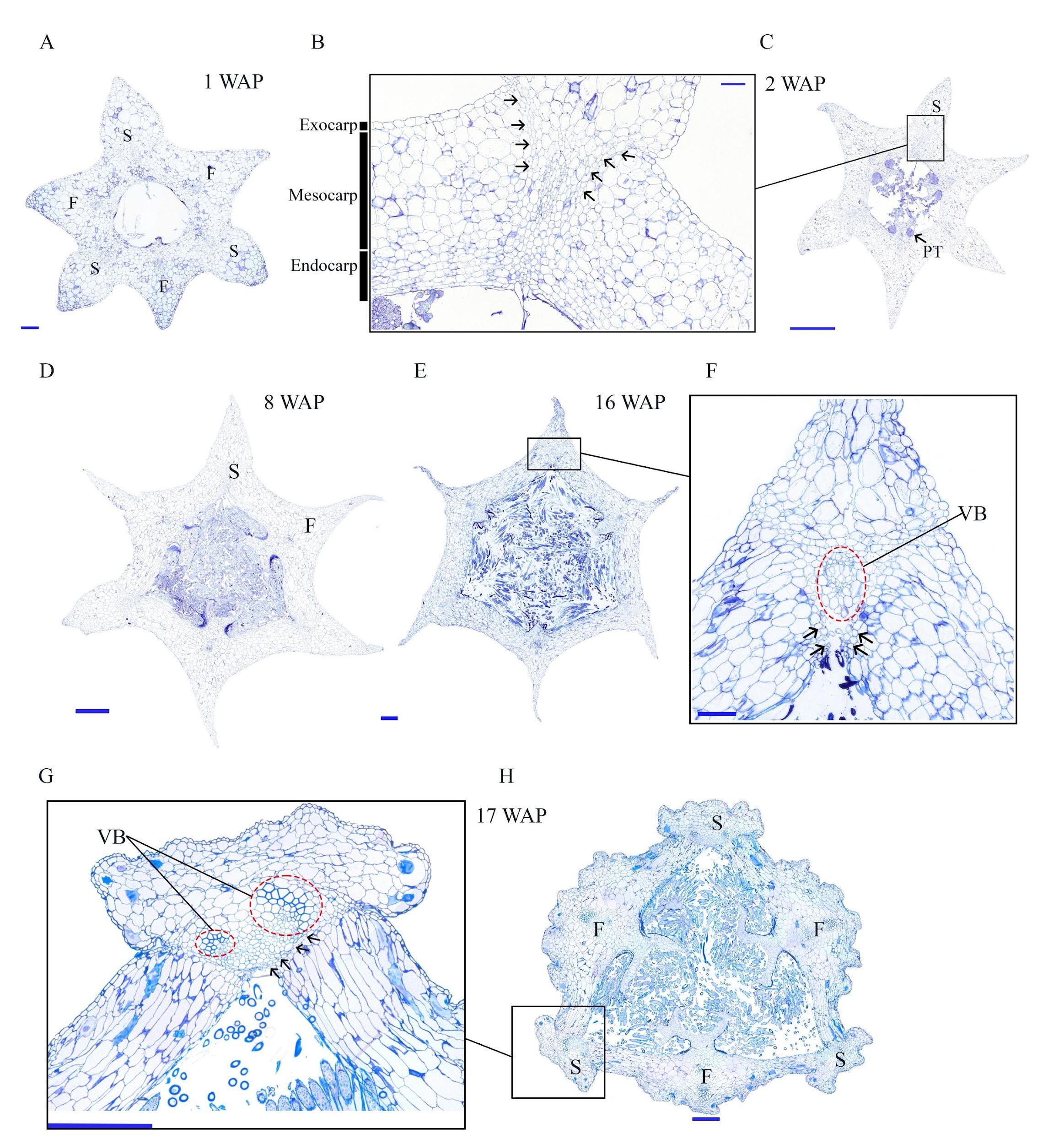


**S1 Fig. Cross-sections of developing *E. pusilla* (A-F) and *P. equestris* fruits (G-H), embedded in LR White and stained with toluidine blue.** (**A**) Fruit 7 days after pollination (DAP). (**B**) Magnified part of the sterile valve at 2 weeks after pollination (WAP). Arrows indicate the dehiscence zone. Black boxes the exo-, meso- and endocarp layer. (**C**) Fruit of 2 WAP. (**D**) Fruit of 4 WAP **(E**) Fruit of 16 WAP. (**F**) Magnified part of the sterile valve at 16 WAP. (**G)** Fruit of 120 DAP. (**G)** Magnified part of the sterile valve at 120 DAP; cell with thick blue stained wall part of vascular bundle (VB) indicates sclerenchyma fiber cap. Black arrows indicate the dehiscence zone. F, fertile valve; S, sterile valve; PT, pollen tube. Scale bar (C,K) = 0.2 mm, (D) = 0.1 mm, (E–I) = 1 mm, (J,G, H) = 0.5 mm. [Figs A-F from [Dirks‑Mulder et al. (2019)](https://sciwheel.com/work/citation?ids=9491560&pre=&suf=&sa=0&dbf=1); Figs G-H captured by Dewi Pramanik].
