# Supplementary material for: Evolution and development of fruits of Erycina pusilla and other orchid species
Source: PLoS One. 2023 Oct 10;18(10):e0286846. doi: 10.1371/journal.pone.0286846 (PMC10564159; doi:10.1371/journal.pone.0286846)
Supplement: S2 Fig — (DOCX) [file pone.0286846.s002.docx]

**
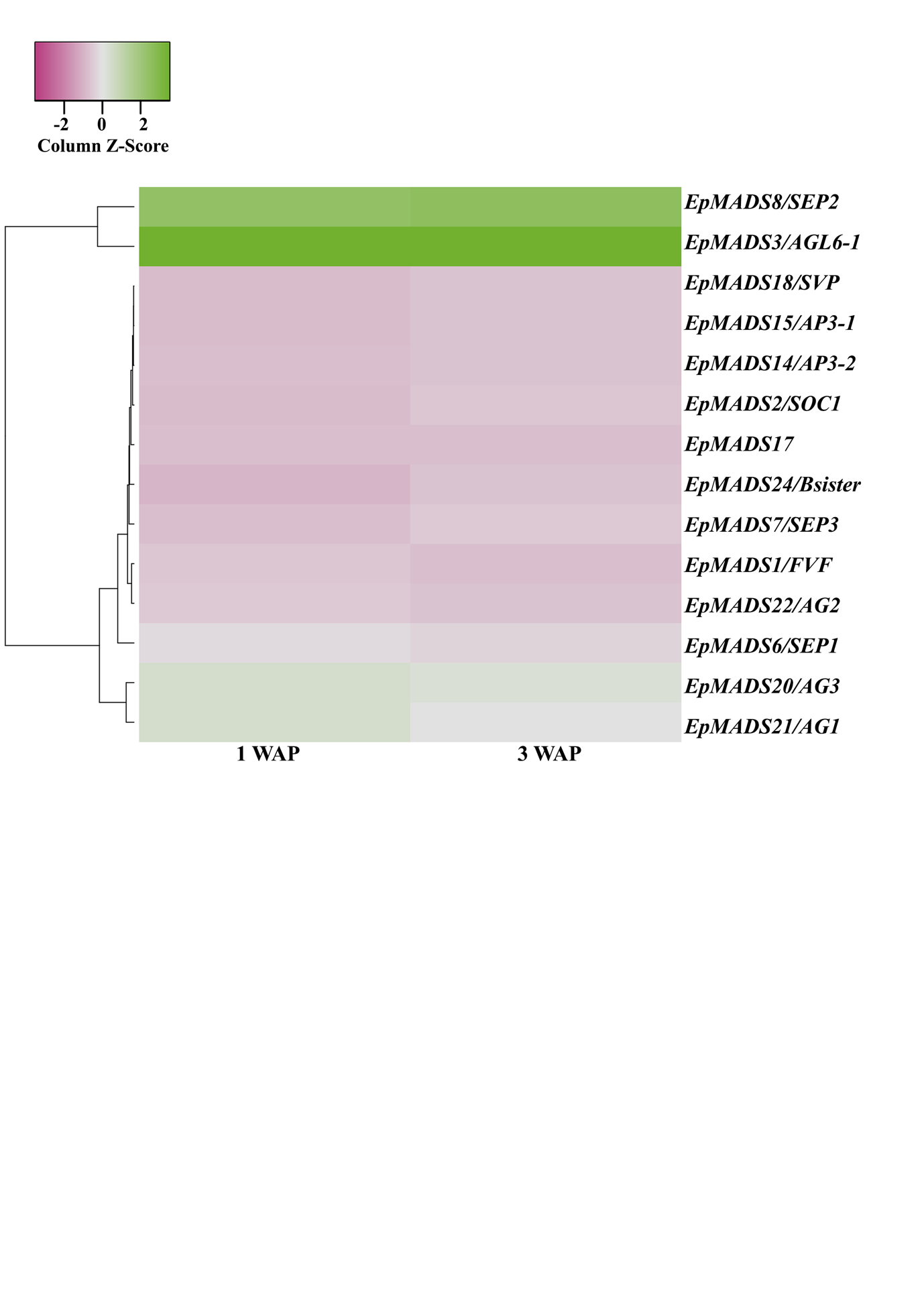
**

**S2 Fig.** **RNA-seq heatmap (TPM) of MICK-MADS-box transcription factors transcriptionally active in one week after pollination (1 WAP) and three weeks after pollination (3 WAP) fruits of *E. pusilla*.**
